# Efficacy of extended infusion of β-lactam antibiotics for the treatment of febrile neutropenia in haematologic patients: protocol for a randomised, multicentre, open-label, superiority clinical trial (BEATLE)

**DOI:** 10.1186/s13063-020-04323-0

**Published:** 2020-05-18

**Authors:** J. Laporte-Amargos, C. Gudiol, M. Arnan, P. Puerta-Alcalde, F. Carmona-Torre, M. Huguet, A. Albasanz-Puig, R. Parody, C. Garcia-Vidal, J. L. del Pozo, M. Batlle, C. Tebé, R. Rigo-Bonnin, C. Muñoz, A. Padullés, F. Tubau, S. Videla, A. Sureda, J. Carratalà

**Affiliations:** 1 Infectious Diseases Department Hospital Universitari Bellvitge, Institut d’Investigació Biomedica de Bellvitge (IDIBELL), Feixa Llarga SN, 08907, L’Hospitalet de Llobregat, Barcelona, Spain; 2 University of Barcelona, Barcelona, Spain; 3 Spanish Network for Research in Infectious Disease (REIPI), Instituto de Salud Carlos III, Sevilla, Spain; 4 Institut Català d’Oncologia, IDIBELL, Barcelona, Spain; 5 Clinical Haematology Department, Institut Català d’Oncologia-Hospitalet, IDIBELL, Barcelona, Spain; 6 Infectious Diseases Department, Institut d’Investigacions Biomèdiques August Pi i Sunyer (IDIBAPS), Hospital Clínic i Provincial de Barcelona, Barcelona, Spain; 7 Infectious Diseases Department, Clínica Universidad de Navarra, Universidad de Navarra, Pamplona, Spain; 8 Clinical Haematology Department, Institut Català d’Oncologia-Badalona, Institut Josep Carreras (IJC), Hospital Germans Trias i Pujol, Barcelona, Spain; 9 Biostatistics Unit, IDIBELL, Barcelona, Spain; 10 Clinical Laboratory Department, Hospital Universitari Bellvitge, IDIBELL, Barcelona, Spain; 11 Pharmacy Department, Clinical Trial Unit, Institut Català d’Oncologia, IDIBELL, Barcelona, Spain; 12 Pharmacy Department, Hospital Universitari Bellvitge, IDIBELL, Barcelona, Spain; 13 Microbiology Department, Hospital Universitari Bellvitge, IDIBELL, Barcelona, Spain; 14 Clinical Pharmacology Department, Hospital Universitari Bellvitge, IDIBELL, Barcelona, Spain

**Keywords:** Febrile neutropaenia, β-lactam antibiotics, Cefepime, Piperacillin-tazobactam, Meropenem, Extended infusion, Randomised controlled trial

## Abstract

**Background:**

Febrile neutropaenia (FN) is a very common complication in patients with haematological malignancies and is associated with considerable morbidity and mortality. Broad-spectrum antipseudomonal β-lactam antibiotics (BLA) are routinely used for the treatment of cancer patients with FN. However, the clinical efficacy of BLA may be diminished in these patients because they present with pathophysiological variations that compromise the pharmacokinetic (PK) parameters of these antibiotics. Optimised administration of BLA in prolonged infusions has demonstrated better clinical outcomes in critically ill patients. However, there is a paucity of data on the usefulness of this strategy in patients with FN.

The aim of this study is to test the hypothesis that the administration of BLA would be clinically more effective by extended infusion (EI) than by intermittent infusion (II) in haematological patients with FN.

**Methods:**

A randomised, multicentre, open-label, superiority clinical trial will be performed. Patients with haematological malignancies undergoing chemotherapy or haematopoietic stem-cell transplant and who have FN and receive empirical antibiotic therapy with cefepime, piperacillin-tazobactam or meropenem will be randomised (1:1) to receive the antibiotic by EI (during half the time of the dosing interval) in the study group, or by II (30 min) in the control group.

The primary endpoint will be clinical efficacy, defined as defervescence without modifying the antibiotic treatment administered within the first 5 days of therapy. The primary endpoint will be analysed in the intention-to-treat population. The secondary endpoints will be pharmacokinetic/pharmacodynamic (PK/PD) target achievement, bacteraemia clearance, decrease in C-reactive protein, overall (30-day) case-fatality rate, adverse events and development of a population PK model of the BLA studied.

**Discussion:**

Data on the usefulness of BLA administration in patients with FN are scant. Only three clinical studies addressing this issue have been published thus far, with contradictory results. Moreover, these studies had some methodological flaws that limit the interpretation of their findings. If this randomised, multicentre, phase IV, open-label, superiority clinical trial validates the hypothesis that the administration of BLA is clinically more effective by EI than by II in haematological patients with FN, then the daily routine management of these high-risk patients could be changed to improve their outcomes.

**Trial registration:**

European Clinical Trials Database: EudraCT 2018–001476-37.

ClinicalTrials.gov, ID: NCT04233996.

## Background

Febrile neutropaenia (FN) is a very common complication in patients with haematological diseases and is associated with considerable morbidity and mortality. Neutropaenia increases the susceptibility of patients to infections, which are more frequent and more severe depending on the degree and duration of the neutropaenia. However, only 30% of patients with FN develop a clinically or microbiologically documented infection, and only 20% develop bacteraemia. The epidemiology of bacteraemia in cancer patients has changed in recent decades, with an increase in Gram-negative bacilli, and more importantly, an emergence of multidrug antibiotic resistance among these organisms [1, 2]. This is of particular concern because adequate empirical antibiotic treatment is associated with increased survival rates in patients with FN [3].

β-lactam antibiotics (BLA) are the most widely used antibiotics in the routine management of FN. Therefore, optimising the use of BLA is essential to improve the prognosis of these patients. One way to optimise the use of these drugs is to administer them by extended infusion (EI) or continuous infusion (CI). This approach allows free (unbound) concentrations of the drug to be maintained above the minimum inhibitory concentration (MIC) of the pathogen during the dosing interval (*f*T > MIC) [4, 5]. Studies seem to show improved outcomes in terms of microbiological eradication, clinical success and mortality when critically ill patients achieve this pharmacokinetic/pharmacodynamic (PK/PD) target [6–9]. Moreover, two recent meta-analyses have shown an association between prolonged infusion of BLA and lower mortality rates [10, 11]. Similar results have also been observed in a non-randomised prospective study in cirrhotic patients with bloodstream infections, in whom prolonged infusion of BLA was associated with improved survival [12]. Nevertheless, earlier studies addressing this issue have mainly focussed on the general non-severely immunocompromised population.

Patients with FN are similar to critically ill patients in terms of the intra- and inter-individual variability of PK parameters, mainly due to an increased volume of distribution and an increased renal clearance [13–15]. BLA PK parameters are widely affected by these pathophysiological changes. In fact, it has been suggested that in patients with FN, some BLA administered by II may not achieve desirable PK/PD parameters [16–18]. Moreover, the PK/PD therapeutic target is still uncertain in neutropaenic patients, and further studies are required to validate the magnitude of the optimal PK/PD index.

Regarding the clinical impact of optimised BLA, data on neutropaenic patients are limited and restricted to certain antibiotics. A prospective, randomised pilot study that analysed cefepime administered by EI and II found no significant clinical differences between the two strategies [19]. However, it should be noted that septic patients were excluded from the analysis, despite being the subgroup that would most benefit from EI. A retrospective study with 164 patients found clinical efficacy with the use of meropenem by EI compared to II [20]. Similarly, a recent single-centre, randomised clinical trial found that EI was associated with superior treatment outcomes compared with II, and the benefit was greatest for patients with pneumonia [21]. Nevertheless, the study had some methodological flaws, and no PK studies were performed to support the clinical results [22].

In the current era of widespread antimicrobial resistance, and due to the lack of new antibiotics, the use of optimised administration of BLA may play an important role in the management of infections in cancer patients. For instance, it may prevent the emergence of resistance [23] and may be a better approach for treating hard-to-treat infections [24].

This randomised clinical trial aims to test the hypothesis that the administration of BLA would be clinically more effective by EI than by II in haematological patients with FN. Patients with FN may benefit from the clinical impact of this strategy, reducing the duration of fever, the global antibiotic consumption and mortality rates. A PK/PD study will also be performed to assess the correlation with the clinical results.

## Hypothesis

The administration of BLA by EI would be clinically more effective than by II in haematological patients with FN.

In haematological patients with FN, the administration of BLA by EI would be superior to II in reaching the PK/PD target, clearing up bacteraemia more quickly and decreasing inflammatory biomarkers. Both strategies would be equally safe.

## Trial design

The BEATLE study is a phase IV, randomised, multicentre, open-label, superiority clinical trial to determine the clinical superiority of EI versus II administration of BLA used as empirical treatment in patients with FN. The BLA studied will be cefepime, piperacillin-tazobactam and meropenem. Patients will be randomised (1:1) at FN onset to receive BLA by EI or II. The intervention will last 5 days.

The manuscript has been prepared in accordance with the Standard Protocol Items: Recommendations for Interventional Trials (SPIRIT) [25], Additional file 1. The study will be conducted in accordance with Good Clinical Practice (IVH-E6).

## Methods

### Setting

The study will be conducted in four university hospitals in Spain. The reference site will be Institut Català d’Oncologia L’Hospitalet (ICO Hospitalet) – Hospital Universitari Bellvitge. The other three participating sites will be Hospital Clinic de Barcelona (Barcelona), Hospital Germans Trias i Pujol (Barcelona) and Clínica Universidad de Navarra (Pamplona).

### Study population and screening of cases for recruitment

This study will include adult patients with haematological malignancies admitted to the haematology wards who are undergoing chemotherapy or haematopoietic stem-cell transplant (HSCT) and experience FN; participants will receive empirical antibiotic therapy with one of the BLA studied.

#### Inclusion criteria

Adult patients (age ≥ 18 years) undergoing chemotherapy or HSCT who present with FN (defined as axillary temperature ≥ 38.0 °C and < 500 neutrophils/mm^3^ or < 1000 expected to drop within 24–48 h), and who receive empirical antibiotic therapy with cefepime, piperacillin-tazobactam or meropenem, in monotherapy or in combination with another antibiotic.

#### Exclusion criteria

1. Allergy to the study drugs
2. Patient receiving systemic antibiotic treatment (except for prophylaxis) at the time of FN onset
3. Absence of fever
4. Patients with epilepsy
5. Severe renal impairment (defined as creatinine clearance rate by MDRD/CKD-EPI < 30 mL/min)
6. Previously enrolled patients in whom the time between inclusion and the current episode is less than 5 weeks
7. Previously enrolled patients without current resolution of the first episode

### Intervention

Patients admitted in the haematology wards of the participating centres, receiving chemotherapy or HSCT will be followed up daily by the attending physicians. The study investigators will explain the study to the patients who meet the inclusion criteria, and will ask them to sign the informed consent. At the FN onset, patients will be randomised to receive EI or II (1:1). The BLA will be chosen according to the clinical criteria of the attending physician. Once randomised, the first BLA dose will be administered within 30 min in all patients. The second dose will be administered according to the randomisation group. In the control group (II) the BLA will be administered within 30 min at the usual doses recommended for neutropaenic patients (cefepime 2 g/8 h, piperacillin-tazobactam 4 g/6 h and meropenem 1 g/8 h), adjusted according to renal function (Table 1). In the study group, the BLA will be administered at the same doses but by EI (the infusion time will be equal to half the time of the dosing interval). Episodes of FN will be classified as ‘microbiologically documented infection’ (with or without bacteraemia), ‘clinically documented infection’ (without microbiological isolation), ‘fever of unknown origin’ or ‘non-infectious fever’.

**Table 1:** β-lactam antibiotic adjustment in renal impairment

*β-lactam antibiotic*	*Creatinine clearance* *(mL/min)*	*Dose*	*Frequency*
Piperacillin-tazobactam	30-40	4 g/0.5 g	Every 8 hours
Cefepime	30-50	2 g	Every 12 hours
Meropenem	30-50	1 g	Every 12 hours

Patients will remain in the study until completing a total of 5 days of antibiotic treatment, or until one of the following events occur: discontinuation of BLA based on clinical criteria, severe adverse event (SAE) or death. If any microorganism is isolated, in vitro susceptibility tests will be performed and antibiotic treatment will be targeted accordingly. If the BLA needs to be switched to another BLA also included in the study due to a lack of in vitro activity, the patient will remain in the study. Measurements and visits will start along with the initiation of the second BLA. If the targeted therapy is an antibiotic not included in the study, the patient will be withdrawn from the study. All patients will receive follow-up for 30 days after initiation of the antibiotic treatment.

The determination of plasma BLA concentrations will not influence the dosage, indication or duration of the BLA administered during the study in any case.

The participant timeline during the study is described in Table 2.

**Table 2:**
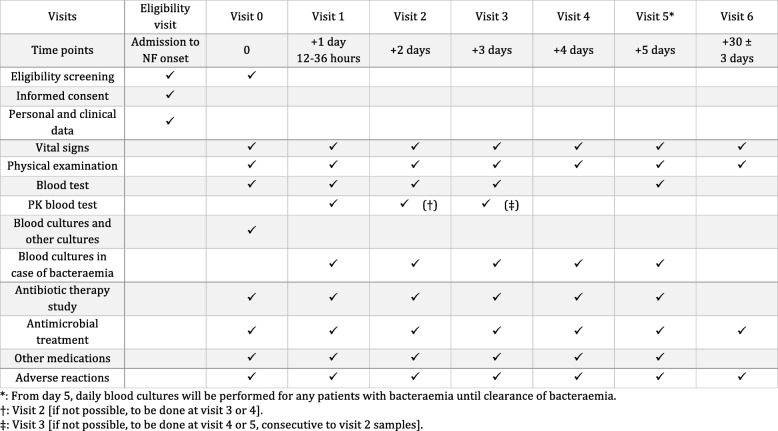
Participant timeline of the study

### Discontinuation criteria

1. Withdrawal of consent
2. Non-compliance with the inclusion or exclusion criteria
3. Serious adverse reaction
4. Protocol violation

In accordance with the current revision of the Declaration of Helsinki and the applicable regulations, a patient has the right to withdraw from the study at any time and for any reason, without this adversely affecting the medical care provided by the patient’s physician. The withdrawal of full consent from a study means that the patient does not wish to continue participating in the study. Any patient withdrawing their consent will be removed from the study treatment and/or observations immediately after the date the patient requests it.

### Outcome measures

#### Primary endpoint

The primary study endpoint is clinical efficacy, measured by the time to defervescence and no need for antibiotic change. To assess this endpoint the rate of patients with defervescence with no change to the antibiotic treatment within the first 5 days of therapy will be calculated. Defervescence will be considered to be an axillary temperature < 37.5 °C for 24 h, assessed at three consecutive time points, with no new documented fever. For this purpose, axillary temperature will be assessed three times daily by the attending nurse. Time (in hours) to defervescence will also be analysed. In addition, prescribed antibiotics will be reviewed every day and recorded in the Electronic Case Report Form (E-CRF) by the participating investigators at each site to estimate the number of patients who will not require an antibiotic change.

The primary endpoint will be analysed by an investigator who will be blind to the treatment group of each patient.

#### Secondary endpoints

Secondary endpoints will be assessed as follows:

1. PK targets: number of patients in whom the free-antibiotic concentration remains above the MIC of the suspected or isolated microorganism for 50%, 75% and 100% of the dosing interval. The same target will also be assessed, taking into account the number of times per patient. Actual MIC values will be used to assess the PK target in those patients with an isolated pathogen. Otherwise, a surrogate MIC will be inferred from the EUCAST database as the highest MIC in the susceptible range for *Pseudomonas aeruginosa* [26]. For this purpose, the nurse in charge of each patient will take blood samples to determine plasma BLA concentrations at 50 and 100% of the dosing interval at visits 1, 2 and 3, and at 75% of the dosing interval at visit 2. The samples will be frozen and subsequently analysed in the biochemistry laboratory at the reference site
2. Bacteraemia clearance: time in days until bacteraemia clearance. In patients with bacteraemia, daily blood cultures will be performed by the nurse in charge of each patient until bacteraemia clearance
3. Inflammatory biomarkers: time in days to normalise or decrease > 50% the peak value of C-reactive protein (CRP), and the number of patients who achieve this objective. Blood samples for CRP determination will be drawn at visits 0, 1, 2, 3 and 5, and will be analysed at each site
4. Overall (30-day) case-fatality rate: all patients included in the study will receive follow-up for 30 days after the end of the intervention by the participating investigators. Death by any cause will be recorded in the E-CRF
5. Population PK analysis to characterise cefepime, piperacillin-tazobactam and meropenem PKs in haematological patients with FN: a population PK model will be developed and validated, using concentration-time data obtained from the blood samples of patients at the reference site (ICO Hospitalet). For each antibiotic, a simultaneous analysis of all concentration-time data will be performed by the population approach using the non-linear mixed-effect models implemented in NONMEM 7.4 (Icon development). The model will allow us to identify the clinical covariables that modify the PK parameters. Monte-Carlo simulations will be performed to determine the suitability of different dosing regimens assessed by the probability of target attainment. The validation cohort will include data obtained from patients at the other sites
6. Adverse events (AEs): all AEs considered not related to the haematological disease or its treatment and all SAEs will be recorded in the E-CRF by the participating investigators. AEs will be identified and classified according to the severity and potential relationship with the BLA and assessed during the intervention (until visit 5). Afterwards, any AEs related to BLA administration and any SAEs until visit 6 (30 days from the beginning of the intervention) will be recorded

### Data collection and management

All data will be collected by the clinical investigators at each participating site and entered in the E-CRF. The collected data will be age, sex, height, weight, type of underlying disease, other comorbidities, date of hospital admission and discharge, HSCT, type and date of HSCT, immunosuppressive therapy, concomitant medication (particularly other systemic antibiotics, prophylactic antimicrobials, fluids, antipyretics and granulocyte-colony stimulating factor), duration of neutropaenia, vital signs, duration of fever, clinical examination, blood tests and microbiological results.

### Microbiological studies

Blood cultures will be drawn at the onset of fever. In patients with bacteraemia, daily blood cultures will be taken until clearance. Other cultures will be collected according to the usual practice and clinical criteria.

Cultures will be processed in the microbiology laboratories at each participating site according to the usual techniques (BACTEC Becton Dickinson, BioMerieux, etc.). The microorganisms isolated will be identified according to the usual methods (conventional method, MALDI-TOF, etc.). Antibiotic susceptibility will be studied by the disc-diffusion method and/or by microdilution method. In addition, the exact MIC of the antibiotics administered will be determined by a quantitative method, using E-test and/or adequate microdilution plates.

### Sample collection for PK/PD analysis

Plasma BLA concentrations will be determined seven times. At visit 1 (which should be performed between 12 and 36 h from the start of the study antibiotic treatment), the concentrations will be analysed pre-dose (through concentrations or C_min_) and then mid-way through the dosing interval (C_50_). Subsequently, the C_min_, C_50_ and the concentration at the 75% of the dosing interval (C_75_) will be analysed at visit 2 (if not possible, this will be done at visit 3 or 4). Last, C_min_ and C_50_ will be analysed at visit 3 (or if not possible, at visit 4 or 5, consecutive to the visit-2 samples).

At the reference site, intensive sampling will be carried out at visit 2. The schedule according to drug, method of administration and frequency is explained in Tables 3 and 4.

**Table 3:** Sampling time in the study group (β-lactam antibiotic administration by extended infusion (EI)) according to the frequency of administration. Time expressed in minutes from the start of drug administration

	Frequency of administration	C_min_	C_max_ (C_50)_	C_max + 30_	C_max + 60_	C_75_
EI	6h	-10	180	210	240	270
	8h	-10	240	270	300	360
	12h	-10	360	390	420	540

**Table 4:**
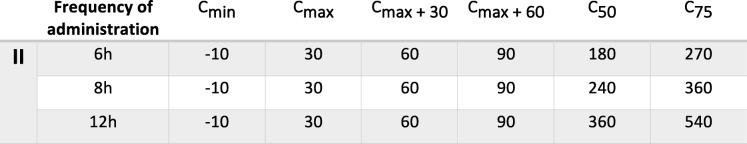
Sampling time in the control group (β-lactam antibiotic administration by intermittent infusion (II)) according to the frequency of administration. Time expressed in minutes from the start of drug administration

A 5-mL blood sample will be centrifuged at 2000 g for 10 min at 4 °C, and the supernatant will be aliquoted in 1.5-mL microcentrifuge tubes and and stored at − 80 °C until analysis. Samples will be sent in dry ice to Bellvitge University Hospital for bioanalysis.

Total plasma BLA concentrations will be analysed using a previously validated method based on ultra-high-performance liquid chromatography coupled to tandem mass spectrometry [27]. Protein binding of 30%, 19% and 2% will be applied for piperacillin, cefepime and meropenem, respectively, to the concentrations determined to estimate the unbound fraction [28, 29].

### Randomisation

Enrolled patients who sign the informed consent will be randomised to receive BLA by either EI or II (1:1). A centralised electronic computer randomisation schedule will be developed by the Biostatistics Department of the reference site. To ensure that an equal number of participants is assigned to each treatment, patients will be randomised in blocks. The randomisation will be performed in computed-generated variable blocks ranging from four to six, and the investigator will be blind to the size of each block. The code numbers for eligible patients will be assigned in ascending sequential order. The allocation list will be stored at the Biostatistics Department of the reference site. At each participating hospital, patients who provide written informed consent and meet the study criteria will be randomised by investigators, who will obtain the assigned treatment and code number from the computer-assisted web site.

### Sample size

The sample size calculation is based on determining whether the administration of BLA by EI (study group) will be clinically more effective than the administration of BLA by II (control group), for the treatment of FN in haematological patients. Previous data suggest that the clinical efficacy rate in the control group is expected to be 0.45 [20, 21]. A relevant clinical efficacy rate in the study group is expected to be 0.70. Each therapy group will required 75 participants (total of 150) to detect statistically significant differences in clinical efficacy. An alpha risk of 0.05 and a beta risk of 0.2 in a bilateral contrast are assumed, and the sample size allows up to 20% losses to follow-up.

#### Interim analysis

An interim analysis will be carried out once 50% of the cases of each arm are recruited. This analysis will evaluate if the estimated sample size is adequate. The results will be issued by an independent committee of experts not participating in this study.

### Statistical analysis

For the primary endpoint, an intention-to-treat analysis will be carried out. The number of patients in both therapy groups with defervescence and with no change in the antibiotic treatment will be compared by a chi-square test.

For the analysis of secondary endpoints, the between-group difference in the percentage of patients reaching the PK target will be compared. The secondary endpoints are: *(a)* days until bacteraemia clearance, *(b)* time until normalisation or 50% decrease of the initial value of the CRP, and *(c)* days until death by any cause within 30 days, and the incidence of these events will be estimated in each group. Survival analysis methods will be used to compare them using the log-rank test. In addition, the rate of therapeutic failure will be compared.

A population PK model will be developed and validated for each BLA studied in haematological patients with FN, using the concentration-time data obtained from the blood samples. The model will allow us to identify the clinical covariates that modify the PK parameters. Monte-Carlo simulations will be performed to determine the suitability of different dosing regimens assessed by the probability of target attainment.

The AEs and SAEs of the study will be described, and their rates will be compared for each group. The severity, relationship to treatment and resolution will be described.

An analysis by protocol will also be carried out. All study variables will be presented for each group, using descriptive statistics according to the type of variable. The main analysis will be replicated in the following subgroups: patients with ‘microbiologically documented infection’ (with and without bacteraemia), ‘clinically documented infection’, ‘fever of unknown origin’ or ‘non-infectious fever’.

Whenever possible, the estimators of a 95% confidence interval will be included. Statistical significance will be set at a probability level < 0.05. The statistical package used to process the data and carry out the analyses will be R, version 3.6.1 or higher for Windows.

### Monitoring

In compliance with the standards of Good Clinical Practice, the sponsor will monitor the study tasks, following the approved monitoring plan. Among others, the monitoring tasks will include assessment of the correct application of the inclusion and exclusion criteria, assessment of the quality of the data collected, development and reporting of any AEs, and maintenance of patient confidentiality.

The need for a Data Monitoring Committee was waived by the Ethic Committee because of the low impact on the safety of the study patients.

## Discussion

Prolonged infusion of BLA is a simple strategy that has proven to improve survival among certain populations, such as critically ill and cirrhotic patients. Broad-spectrum antipseudomonal BLAs are routinely used to treat cancer patients with FN. Optimised use of these antibiotics could improve the outcomes of these patients, who would benefit from faster defervescence and microbiological eradication, as well as a decrease in overall antibiotic exposure. However, there are few data on the usefulness of this strategy in this high-risk population.

Only three published clinical studies have addressed this issue so far, with contradictory results. A prospective, randomised pilot study compared cefepime administration by II to 33 neutropaenic patients with administration by EI to 30 patients. No significant differences were found in the primary endpoint of defervescence at 72 h or in the secondary endpoints, such as clinical success, in-patient mortality, length of hospital stay or need for additional antibiotics. Although not statistically significant, defervescence seemed to be faster in patients treated with cefepime by EI [19]. The main limitation of this study was the exclusion of patients presenting with sepsis. The presence of sepsis in neutropaenic patients is common and contributes significantly to the pathophysiological changes that compromise the PK/PD parameters of the BLA and its efficacy. Therefore, patients with FN and sepsis are probably the patients who could most benefit from this administration strategy.

A Spanish group compared the clinical efficacy of meropenem by EI versus II in a retrospective observational study involving haematological patients with FN. Data on defervescence, resolution or improvement of signs and symptoms of infection, absence of breakthrough bacteraemia, and absence of additional antibiotics were analysed [20]. The administration of meropenem by EI was found to be clinically more effective, and also achieved defervescence and a significant decrease of CRP more rapidly. The limitations of this study were its retrospective nature, the definition of a combined primary endpoint using a large number of variables and the lack of plasma meropenem determinations.

Last, a recent randomised clinical trial reported better clinical outcomes in patients receiving BLA by EI compared with II, particularly those with pneumonia [21]. Nevertheless, this study had some limitations. First, it was a single-centre study, which may limit extrapolation of the results. Second, the definition used for ‘pneumonia’ was somewhat imprecise, as it included all patients with inflammatory radiological opacities. This is relevant, because pneumonia is the second most common infection reported in this trial. Third, piperacillin-tazobactam was used in > 90% of patients. Therefore, the results should be only interpreted with the use of this antibiotic. Last, no PK/PD determinations were performed to support the study’s findings.

If our randomised, multicentre, phase IV, open-label, superiority clinical trial validates the hypothesis that the administration of BLA by EI is clinically more effective than by II in haematological patients with FN, then this could change the daily routine management of these high-risk patients and, thus, improve their outcomes.

## Trial status

The current protocol is version 3.2, dated 9 January 2020. Patient recruitment started on 12 November 2019 and will be completed by November 2021.

## Supplementary information

**Additional file 1.** SPIRIT 2013 Checklist: Recommended items to address in a clinical trial protocol and related documents.

## Data Availability

All data generated during this study will be included in published article. The datasets will be available from the corresponding author on reasonable request.

## Abbreviations

AE: Adverse event
BLA: β-lactam antibiotics
CI: Continuous infusion
CRP: C-reactive protein
E-CRF: Electronic Case Report Form
EI: Extended infusion
EUCAST: European Society of Clinical Microbiology and Infectious Diseases
FN: Febrile neutropaenia
*f*T > MIC: Time of free drug concentration above MIC
HSCT: Haematopoietic stem-cell transplant
ICO Hospitalet: Institut Català d’Oncologia L’Hospitalet
IDIBELL: Institute of Biomedical Research of Bellvitge
II: Intermittent infusion
MIC: Minimum inhibitory concentration
PD: Pharmacodynamic
PK: Pharmacokinetic
SAE: Serious adverse event
